# Nitric Oxide and Oxidative Stress Changes at Depth in Breath-Hold Diving

**DOI:** 10.3389/fphys.2020.609642

**Published:** 2021-01-07

**Authors:** Danilo Cialoni, Andrea Brizzolari, Michele Samaja, Gerardo Bosco, Matteo Paganini, Massimo Pieri, Valentina Lancellotti, Alessandro Marroni

**Affiliations:** ^1^Environmental Physiology and Medicine Laboratory, Department of Biomedical Sciences, University of Padova, Padova, Italy; ^2^Divers Alert Network (DAN) Europe Research Division, Roseto degli Abruzzi, Italy; ^3^Apnea Academy Research, Padova, Italy; ^4^Department of Health Sciences, Università degli Studi of Milan, Milan, Italy; ^5^Cardiothoracic and Vascular Department, Azienda Ospedaliero-Universitaria Pisana (AOUP), Pisa, Italy

**Keywords:** nitric oxide, breath-hold diving, diving, free radicals, oxidative stress

## Abstract

**Background:**

Several mechanisms allow humans to resist the extreme conditions encountered during breath-hold diving. Available nitric oxide (NO) is one of the major contributors to such complex adaptations at depth and oxidative stress is one of the major collateral effects of diving. Due to technical difficulties, these biomarkers have not so far been studied *in vivo* while at depth. The aim of this study is to investigate nitrate and nitrite (NOx) concentration, total antioxidant capacity (TAC) and lipid peroxidation (TBARS) before, during, and after repetitive breath-hold dives in healthy volunteers.

**Materials and Methods:**

Blood plasma, obtained from 14 expert breath-hold divers, was tested for differences in NOx, TAC, and TBARS between pre-dive, bottom, surface, 30 and 60 min post-dive samples.

**Results:**

We observed a statistically significant increase of NOx plasma concentration in the “bottom blood draw” as compared to the pre-dive condition while we did not find any difference in the following samples We found a statistically significant decrease in TAC at the bottom but the value returned to normality immediately after reaching the surface. We did not find any statistically significant difference in TBARS.

**Discussion:**

The increased plasma NOx values found at the bottom were not observed at surface and post dive sampling (T0, T30, T60), showing a very rapid return to the pre-dive values. Also TAC values returned to pre- diving levels immediately after the end of hyperbaric exposure, probably as a consequence of the activation of endogenous antioxidant defenses. TBARS did not show any difference during the protocol.

## Introduction

Breath-hold (BH)-diving is the first recorded type of underwater activity, practiced since ancient times for commercial (sponge diving and pearl harvesting) or military purposes, and more recently for leisure purposes (Ashcroft, 2002). The most striking recognized physiological adaptation feature during BH-diving is related to the higher than normal hydrostatic pressure, that increases by one atmosphere every 10-m depth. This necessarily reflects into complex cardiovascular adaptations, that are collectively termed as “diving response,” which includes important changes such as bradycardia, reduced cardiac output, increased arterial blood pressure, peripheral vasoconstriction and blood gases composition (Heusser et al., 2009).

Nitric oxide (NO) is well-known to be a pivotal molecule responsible for the maintenance of the vascular tone in health and disease (Ignarro et al., 1999; Napoli and Ignarro, 2009). NO is a free radical because of the unpaired electron in the outer orbit. In biology, NO is also an important signaling molecule involved in several physiological and pathological processes, especially vasodilation (Rand, 1992; Hou et al., 1999). NO formation is catalyzed by a family of NO synthases (NOS), which is composed of at least three isoforms with different intracellular localizations (Moncada and Higgs, 1993). Endothelial NOS (eNOS) plays a key role in modulating the peripheral vascular tone and, consequently, arterial blood pressure (Rand, 1992).

NO plays a pivotal role also in subjects exposed to high hydrostatic pressures (Theunissen et al., 2013a), and recent measurements taken in scuba divers at −40 m depth showed remarkable increases in the plasma concentrations of NO derivatives (Cialoni et al., 2019).

As a matter of fact, direct measurement of blood NO is not always performed (Moller et al., 2019), but the level of NO derivatives such as nitrates and nitrites (NOx) (Van Vliet et al., 1997), products of NO oxidation in blood and in tissues (Rand, 1992), may nevertheless provide an indirect estimation of available NO in the circulation.

Being NO a free radical with short half-life ranging around 0.05–1.8 ms (Rand, 1992; Hou et al., 1999), it is also expected that higher- than-normal NO levels in the circulation may combine with superoxide anions (O2^⋅–^) giving rise to aggressive reactive nitrogen species (RNS) such as peroxynitrite (ONOO^–^) that, besides inactivating some NO synthases (Munzel et al., 2010), triggers the formation of reactive oxygen species (ROS) leading to oxidative stress. Oxidative stress may also be favored by the high pO2 due to high pressure, which increases the amount of free O2 that may favor mitochondrial uncoupling and represent an independent source of ROS (Ottolenghi et al., 2020).

Oxidative stress has been investigated in diving (Brubakk et al., 2005; Obad et al., 2010), both in self-contained underwater breathing apparatus diving (SCUBA) (Theunissen et al., 2013a) and BH-diving (Theunissen et al., 2013b). Recent confirmations suggest a primary role of oxidative stress increase as the cause of endothelial dysfunction (Cai and Harrison, 2000). LC-mass spectrometry methods represent the golden standard laboratory measurement to investigate blood oxidation markers in terms of sensitivity and specificity but are expensive and require specific/complex instrumentation (Frijhoff et al., 2015). On the other hand, a less specific spectrophotometric method to measure the ROS/RNS produced by cellular metabolism, environmental factors and the balance between oxidant and antioxidant agents, is to investigate the total plasma antioxidant capacity (TAC) (Bartosz, 2010; Pinchuk et al., 2012). One of most common tests to evaluate TAC is the Trolox equivalent antioxidant capacity (TEAC) assay with modifications mainly based on period of time used for measurement, and radicals formed (Miller et al., 1993; Re et al., 1999; Erel, 2004). Other methods to investigate TAC are ferric reducing antioxidant power (FRAP) and oxygen radical absorbance capacity (ORAC) assays (Cao et al., 1993; Benzie and Strain, 1996).

A further way to investigate oxidative stress is the evaluation of lipid peroxidation that proceeds via a free radical chain mechanism producing many end products (short-chain alcohols, aldehydes and ketones), with aldehydes being prominent among them. One of the most studied aldehydes is malondialdehyde (MDA) as it is associated with off-flavors and aromas in meat products (Fernandez et al., 1997) as well as being a marker of oxidative damage in physiological systems (Del Rio et al., 2005). There are numerous methods for measuring MDA, including GC, HPLC (Del Rio et al., 2005), and capillary electrophoresis (Wilson et al., 1997), but, by far, the most common method is through reaction of MDA with thiobarbituric acid (TBA) to produce a pink-colored dimeric compound. Despite the non-specificity of the method, TBARS assay finds wide application in food analysis (Guillen-Sans and Guzman-Chozas, 1998) and in studies on human health and disease, including cardiovascular disease (Okauchi et al., 2011), obesity and diabetes (Furukawa et al., 2004). A panel of oxidative stress markers within a study aimed at investigating the effects of hyperoxia in anesthesia, found that MDA, the main end product of the peroxidation of polyunsaturated fatty acids, as measured by the TBARS assay, may represent the best marker to assess the pro-oxidant/antioxidant equilibrium in a surgical context (Ottolenghi et al., 2019). For this reason, although not representing the cutting edge of sensitivity, TAC and TBARS assay still represent a reasonable compromise in terms of costs and reliability.

Available literature in this field, however, did not reveal profound changes in neither NOx or oxidative stress (Theunissen et al., 2013a, b; Radojevic-Popovic et al., 2015): perhaps this may be due to the fact that the blood samples usable for this were obtained on the surface before or immediately after the dive, when the changes were masked by the rapidity with which the organism responds to the return to normal conditions.

The aim of this study is to examine critically whether data related to NO and oxidative stress must be obtained during the dive itself, and not simply at the return to normal sea level conditions, investigating NOx concentration and oxidative stress markers before, during, and after repetitive BH-dives in healthy volunteers.

Given the ephemeral and rapidly transient nature of NO metabolism, any study not evaluating its changes over the time of exposure to rapid environmental and physiological changes may not show the biochemical “adaptation continuum” of NO metabolism to external stress conditions; the novelty of this study is having for the first time assured that the collection of blood samples could be simultaneous with the exposure to underwater hyperbaric exposure of human divers.

## Materials and Methods

### Subjects

We recruited 14 healthy BH-divers, who were investigated during a series of deep dives at “Y-40 The Deep Joy” pool (Montegrotto Terme, PD, Italy). Subjects were asked to avoid food rich in NOx, such as red meat (Lundberg, 2009) and leafy green vegetables (Lundberg and Govoni, 2004), and intense exercise during the 48 h before the experiment. All divers were informed about the risks and benefits of this study, read and signed a specific informed consent form before the experiment, and gave personal anthropometric data. The study was conducted in accordance with the Helsinki Declaration and was approved by the Ethical Committee of the Università degli Studi di Milano, Italy (Aut. No. 37/17).

The selected volunteers are labeled “expert” because they are affiliated to the “Apnea Academy” Training Agency as instructors or high-level BH-divers, and are able to reach −42 m depth in variable weight, 4 min static apnea (at the surface), 75 m dynamic BH-diving (horizontal) in a swimming pool (distance).

The exclusion criteria were: history or clinical evidence of hypertension, cardiac, pulmonary, or any other significant disease; any acute illness during the 15 days before the experiment; use of aspirin, paracetamol, or other anti-inflammatory drugs in the 7 days before the experiment; compressed-gas diving during the 30 days before the test.

The dives were performed in teams of two subjects each as per the no-limits BH-diving technique (using a dedicated machine to facilitate descent and ascent).

All the subjects performed a gradual warm-up, with a determined number of dives at increasing depth: 1st dive at −15 m; 2nd dive at −25 m; 3rd dive at −35 m. The volunteers were however allowed to adapt the warm-up protocol to their needs. When ready, they made one dive to the bottom of the swimming pool (−42 m) where the “bottom blood draw” was obtained. To ensure the safety of the divers, two SCUBA divers were stationed near to the surface during the descending and ascending phases, ready to take action in case of potential troubles.

The following diving parameters were also recorded for each dive, using a free-diving computer (UP-X1 Omersub, Spa, Monza Brianza, Italy): depth; diving time; bottom time; surface interval and numbers of dives. This computer measured and recorded diving data every 2 s.

### Dive Protocol

**FIGURE 1:**
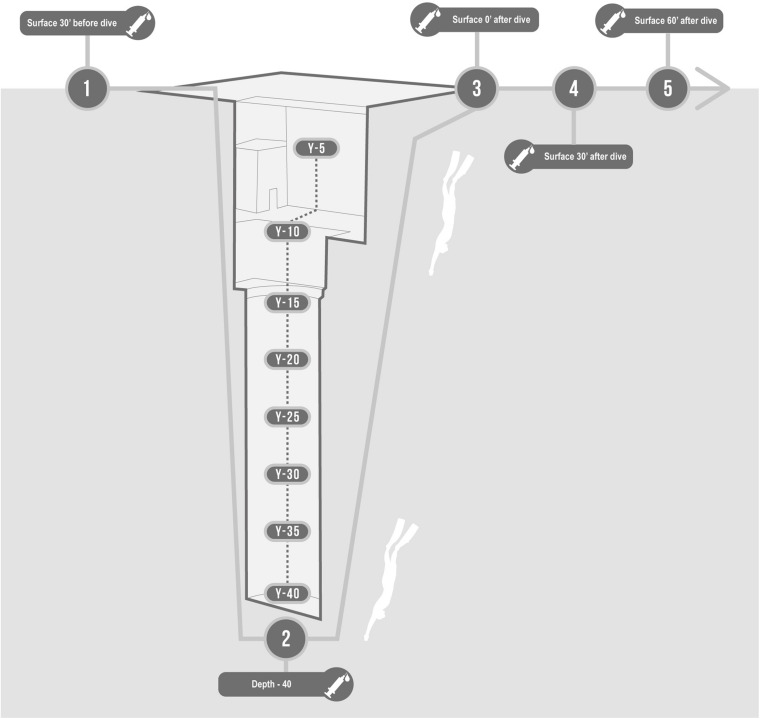
Description of protocol in the swimming pool Y-40. Basal, 30 min before; Bottom, at −42 m; T0, On arrival at the surface; T30, 30 min after; T60, 60 min after.

A 2-way peripheral venous catheter was placed in the antecubital vein before the dive, wrapped in a waterproof bandage, and connected to a 3-way stopcock. We collected blood samples in one EDTA containing tube (Vacutainer, Becton, Dickinson and Company, Franklin Lakes, NJ, United States) per each of the following time steps (Figure 1):

• Basal: 30 min before the start of the warm-up;
• Bottom: at −42 m;
• T0: Immediately on arrival at the surface (head out of the water, while normally breathing);
• T30: 30 min after the deep dive;
• T60: 60 min after the deep dive.

Except for the “basal” test (when the blood sample was taken immediately after the cannula placement), 5 ml of blood was drawn and discarded before collecting the sample in the tube at each step to reduce the interference of blood clotted at the inner side of the cannula. After each sampling, the cannula was flushed with normal saline (NS) to prevent clotting.

At the “bottom” sampling, the stopcock hosted the tube adapter and a 10 ml syringe filled with 5 ml of NS. The subjects waited about 10 s at the bottom, needed to draw 5 ml of blood in the syringe, fill the tube, and then flush the cannula. Finally, each diver brought the tube back to the surface and delivered it to a researcher. No further dives were performed until the conclusion of the experiment with the follow-up at 30 and 60 min. Plasma was separated from the cell component by centrifugation (3,000 rpm for 10 min) immediately after the draw and was refrigerated at −20°C until use, according to the recommendations of Firuzi et al. (2006).

### Plasma NOx Measurement

NOx concentration was measured in the deproteinized plasma. Before the analysis 400 μl of each plasma sample were treated with 400 μl of acetonitrile to precipitate proteins (Romitelli et al., 2007). Briefly, we used a method based on Griess’s reaction as an index of NO concentration (Green et al., 1982), according to Cialoni et al. (2019). Plasma NOx levels were obtained by interpolation of NaNO3 standard (Tsikas, 2005). All the samples were analyzed in duplicates.

### Total Antioxidant Capacity (TAC)

TAC was investigated using Trolox Equivalent Antioxidant Capacity (TEAC) assay, according to Re et al. (1999) with slight modifications. The TEAC Test is based on the reaction with the colored and relatively persistent 2,2′- azinobis (3-ethylbenzothiazoline-6-sulfonic acid) (ABTS^+^^⋅^) radical cation, which has a strong absorption band at 734 nm. The antioxidant activity is defined as the amount of ABTS^+^^⋅^ quenched after a fixed time and is compared with that produced by Trolox (Re et al., 1999). ABTS was dissolved in 10 mM phosphate buffer (pH = 7.4) to give a 14 mM solution. Potassium persulfate was dissolved in water to give a 4.9 mM solution. ABTS radical cation (ABTS^•+^) was produced by mixing the same volumes of ABTS and potassium persulfate stock solutions and allowing the mixture to stand in the dark at room temperature for 16 h before use. Standard solutions of Trolox in a concentration range from 5 to 100 μM were prepared to build the calibration curve. The ABTS^•+^ solution was diluted with phosphate buffer to an absorbance of 0.700 ± 0.02 at 734 nm. After addition of 1.5 ml of diluted ABTS^+^ solution (A734 = 0.700 ± 0.02) to 30 μl of plasma or Trolox standard (final concentration 0.5–20 μM), the absorbance was taken after 20 min of incubation at 25°C, using a Uvikon 931 UV-VIS Spectrophotometer (Northstar Scientific, Bardsey, United Kingdom). The percentage inhibition of absorbance at 734 nm is calculated and plotted as a function of the concentration of antioxidants and of Trolox for the standard reference data. Results were expressed as Trolox equivalents (μmol/l). All the measures were performed in duplicates.

### TBARS Assay

Lipid peroxidation was investigated by TBARS assay, as described by Spirlandeli et al. (2014) with slight modifications. TBARS are low-molecular-weight end products formed during the decomposition of lipid peroxidation products (Tsai and Huang, 2015). TBARS react with thiobarbituric acid to give a colored complex, which can be determined spectrophotometrically (Almroth et al., 2005). For the malonaldehyde (MDA) standard curve, a mother solution of 100 μM MDA tetrabutylammonium salt was prepared daily in 10 mM HCl. Dilutions from mother solution in a concentration range from 0.5 to 50 μM were then performed.

Briefly, 100 μl of plasma or standard solution was added to 1 ml of a mixture containing 15% TCA, 0.38% TBA in 0.25 M HCl. The diluting medium was used as the standard blank. Samples were heated at 95°C for 30 min and then ice-cooled for 2 min. After centrifugation (10,000 rpm for 5 min), 250 μl of centrifuged samples or standard solutions were placed in a 96-well polystyrene microplate and the optical density was read at 532 nm in an EnSight Multimode Plate Reader (PerkinElmer, Waltham, United States). Plasma levels were obtained by interpolation of the standard MDA curve. All the measures were performed in duplicate.

### AGE and BMI Influence

We also investigate if the results obtained underwater, related to NOx, TAC, and TBARS, were influenced by AGE and anthropometric parameters.

### Statistic Analysis

1Data are presented as mean ± standard deviation (SD) for parametric data and median, or range for non-parametric data. To minimize the subject-to-subject variability, data are normalized against the basal value. The D’Agostino and Pearson normality test was used to assume a Gaussian distribution. Then, data were analyzed by either the one-way ANOVA for multiple comparison, or the Friedman test for multiple comparison, respectively, of parametric and non-parametric data. A probability lower than 5% was assumed as the threshold to reject the null hypothesis (*p* < 0.05).

The datasets generated and analyzed during the current study are available from the corresponding author upon request.

## Results

Table 1 shows the characteristics of the subjects recruited for this study. All the subjects respected the warm-up protocol, except for two who requested an adjunctive dive to −25 m to fully achieve correct ear equalization. All the volunteers completed the experiment without Taravana episodes, evidence of pulmonary and/or ear barotraumas or other health trouble.

**TABLE 1:** Summarizes the anthropometric and diving data set.

**Anthropometric data**		
Age (years)	45.5	±10.2
Height (m)	1.78	±0.07
Weight (Km)	78.8	±10.2
BMI (Kg/m^2^)	24.7	±2.6
**Characteristics of dives**		
Mean depth (meters)	29.1	±9.7
Maximum depth (meters)	40.8	±0.6
Diving time (seconds)	118.7	±20.0
Surface interval (seconds)	384.0	±40.7
Numbers of dives	4.1	±0.4

The diving profile showed a mean of dives of 4.1 ± 0.4; a mean depth of 29.0 ± 9.7 m; a mean of diving time of 118.7 ± 20.0 s and a mean surface interval of 384.0 ± 40.7 s. All the subjects reached the bottom of the swimming pool for the blood draw at 42 m (Table 1). Descent time was typically 35–40 s and the blood draw at depth was performed immediately after. Divers spent 30–40 s at maximum depth for sampling, then the ascent phase took between 40 and 45 s.

**FIGURE 2:**
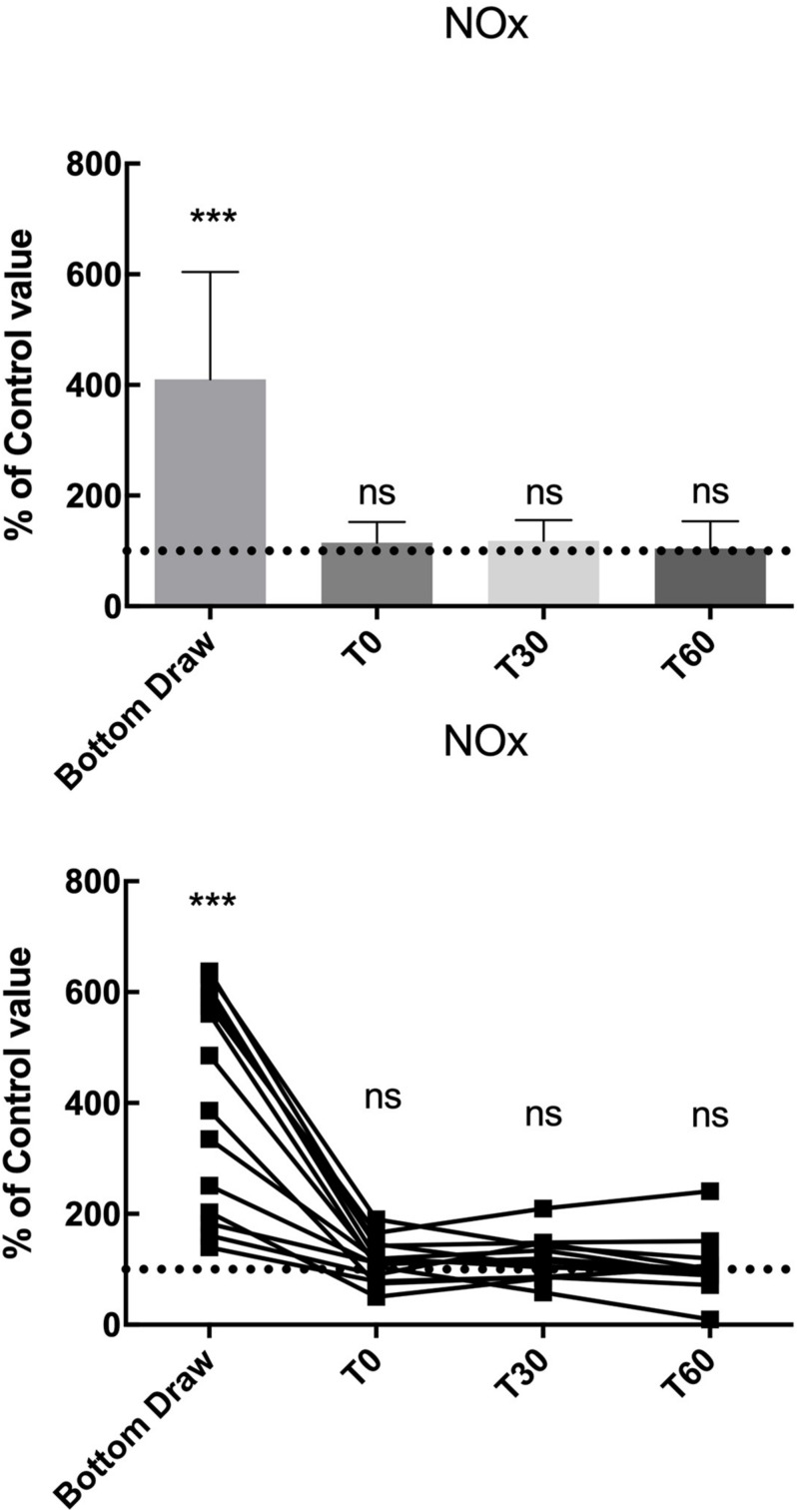
NO_*x*_ changes, compared to the pre value, showed a statistically significant increase at the bottom *p* < 0.0001 while no differences were found in the follow-up. ***Significative differences *p* < 0.001.

We found a statistically significant increase in NOx plasma concentration at the bottom, in terms of percentage of basal value (410.5% ± 194.1; *p* < 0.001), the NOx value returned to normal at T0 and remained unaltered at T30 and T60 (Figure 2).

**FIGURE 3:**
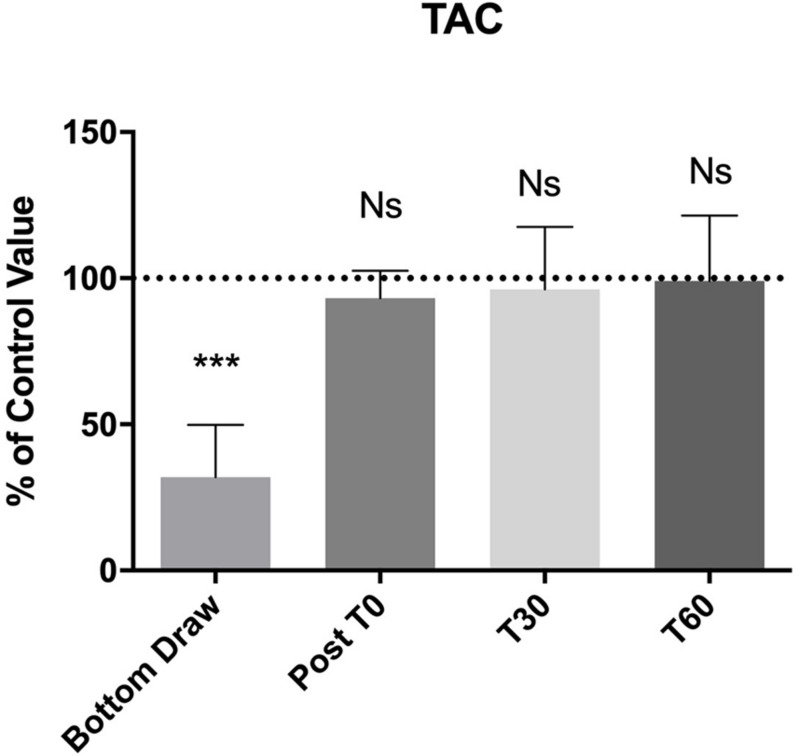
TAC changes, compared to the pre value, showed a statistically significant decreases at the bottom *p* < 0.0001, no differences were found in the follow-up. ***Significative differences *p* < 0.001.

We observed a statistically significant decrease (−60%) in TAC at the bottom (*p* < 0.0001). Also, the TAC value returned to normal at T0 and remained unaltered at T30 and T60 (Figure 3).

**FIGURE 4:**
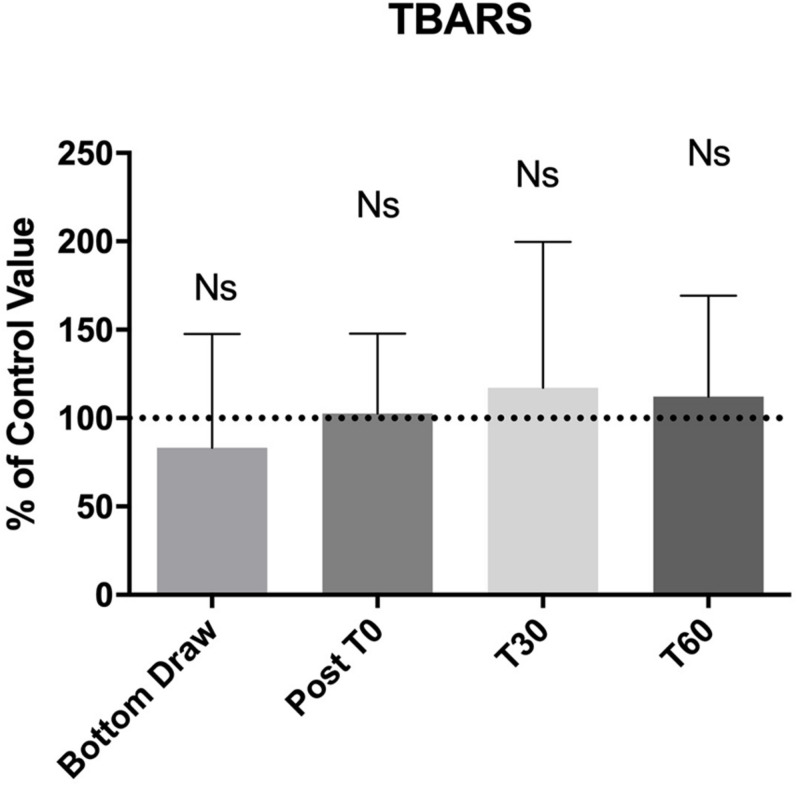
No changes between pre and post BH-diving training session were found about TBARS.

Finally, Figure 4 shows blood TBARS. No appreciable changes were noticed for all draws. We did not find any correlation between the underwater results and AGE or BMI.

## Discussion

As previously explained, NO is a major contributor to vascular adaptations during BH-diving (Theunissen et al., 2013a, b), but the extreme conditions reached by athletes and the intrinsic lability of NO (Moller et al., 2019) make its detection and analysis very difficult in the real environment. On the other hand, oxidative stress is one of the most negative, and studied (Ottolenghi et al., 2020), consequences of hyperbaric exposure (hyperoxia related), and the related free radical study during the underwater phases of a dive is far from easy. Until now, no studies investigated NO and oxidative stress during the underwater phase of a dive, but only before and after the dives (Theunissen et al., 2013a; Sureda et al., 2014).

In this study, we showed that BH-divers experience an increase in NOx, and a decrease in TAC, but is only evident in the blood samples obtained at depth. In the samples obtained at follow-up and after surfacing, no changes were noticed with respect to the baseline condition. No statistical differences were found as concerning TBARS at the bottom.

This is the first study reporting venous blood data in samples obtained at depth during BH-dives. The observation that some data were altered only during depth draws and not at the surface renders it critical that studies aimed at examining human physiology during underwater immersion strictly need the execution of measurements at high hydrostatic pressure conditions.

It is intriguing to note that NOx value immediately returns to normality when reaching the surface. This data is really interesting and seem to indicate that the NOx values found in the post BH-diving test (T0, T30, T60 in our protocol) reflect the NO availability after diving and are not influenced by a NOx accumulation related to diving confirming a very rapid metabolization of the NO derivatives.

It is well known that NO synthesis is catalyzed by a family of NO synthases (NOS), with at least three isoforms with different intracellular localizations (Moncada and Higgs, 1993). Endothelial NOS (eNOS) plays a key role in modulating the peripheral vascular tone and, consequently, arterial blood pressure (Rand, 1992). Thus, alterations in NO production may result in endothelial dysfunctions, and several pathological conditions have been linked to NO reductions that lead to atherosclerosis (Barbato and Tzeng, 2004), thromboembolism (Freedman and Loscalzo, 2003), and even COVID-19-like outcomes (Teuwen et al., 2020). Also in BH-diving an endothelial dysfunction is well studied even if its effect of the development of BH-diving related disease is still unclear (Cialoni et al., 2016; Barak et al., 2020). Anyway, our main our result about the NOx behavior is related to the quickly return to the pre diving value as soon as the BH-diver reached the surface.

In BH-diving, this aspect may be the result of two different mechanisms. First, transient hypoxia, especially experienced in the ascent phase (Bosco et al., 2018, 2020), can reduce NOx due to reconversion to NO. Specifically, NO3 can be reduced to NO2 by several enzymes such as xanthine oxidase (Li et al., 2003) and xanthine oxidoreductase (Jansson et al., 2008). Then, NO2 is reduced to NO by different pathways including hemoglobin (Cosby et al., 2003), myoglobin (Rassaf et al., 2007; Shiva et al., 2007), xanthine oxidoreductase (Godber et al., 2000), or ascorbic acid (Carlsson et al., 2001). These pathways ensure NO production when O2 supply is reduced and the oxygen- dependent NOS enzyme activities are compromised (Giraldez et al., 1997; Ostergaard et al., 2007). The same NO2 reduction pathway has been found in mammalian where NO production from NO_2_ is catalyzed at in part by myoglobin expressed at low levels in their vasculature (Totzeck et al., 2012). Moreover, myoglobin has an high O_2_ affinity making it a fast NO_2_ reductase: data obtained by different species (mammals, fish, birds, reptiles) showed a direct correlation between O_2_ affinity and NO_2_ reductase activity which is related to changes in heme reactivity (Helbo et al., 2013). In trained humans, NO generated by NO_2_ in red blood cells by deoxygenated hemoglobin enhances blood flow to hypoxic exercising muscles and decreases peripheral resistance (Cosby et al., 2003). In diving mammals, a strong adrenergic tone constricts the peripheral circulating system acting to maintain blood pressure to most vital organs with a very low heart rates. NO_2_^–^ derived NO may favor dilation of the large conducting arteries and provide room for the blood volume expelled from the contracting peripheral vessels helping to redistribute blood volume during hypoxia (Fago and Jensen, 2015).

The second mechanism could be related to the diving response characterized by peripheral vasoconstriction (to prevents core hypothermia) and by a shift of interstitial fluid into the plasma for the increase of environmental hydrostatic pressure also causing a reduction of the pulmonary alveolar volume. The resulting of these adaptations is the increase in thoracic blood volume (Arborelius et al., 1972; Norsk et al., 1986) in the underwater phase and, as a consequence, an atrial-received increase of plasma during the ascent phase with inhibition of antidiuretic hormone ADH secretion, to reduce blood volume (Johansen et al., 1992) through the increase in diuresis (Farrell, ; Graveline and Jackson, 1962). Since much of the NO3, the predominant NO oxidation product in blood circulation (Lundberg et al., 2009), is excreted with urine (Lundberg et al., 2008), hyperbaric exposure during BH-diving could reduce NOx through this path.

In our experiment, we also observed a reduction in TAC levels at the bottom that seems to indicate a high use of the antioxidant blood defense in response to oxidative stress in the underwater phase. We evaluated this response against the oxidative stress using TEAC assay. Despite the non-specificity of the method, TEAC assay is one of the most common test to evaluate the TAC changes in biological fluids, showing a good reproducibility (Cao and Prior, 1998; Jansen and Ruskovska, 2013) and avoiding the measure of each antioxidant component that can be labor-intensive and time-consuming, requiring complex, and costly technique (Erel, 2004). In human plasma TEAC measures albumin (that represents 43–53% of the total value), uric acid (representing 33%), ascorbic acid, α- tocopherol, and bilirubin (Miller et al., 1993; Erel, 2004). All these molecules are components of a complex endogenous antioxidant system that takes action against ROS/RNS. On the other hand, TEAC assay doesn’t measure the role of important enzymes such as superoxide dismutase (SOD), glutathione peroxidase (GPx), and catalases (CAT) (Sies, 2007; Bartosz, 2010; Fraga et al., 2014). Therefore, TAC provides a reductionist modeling of an *in vivo* situation and, consequently, caution is needed in the interpretation of data (Costantini, 2011). In BH-divers, the initial hyperoxia, the physical activity and the complex adaptations to increased environmental pressure could justify an increase of free radicals even if TAC, being a non-specific test, does not permit to better understand the nature of this result. Anyway, it is intriguing to note that, also concerning TAC, the plasma level returns to pre-dive value immediately after surfacing, indicating that this effect does not persist after the end of hyperbaric exposure in BH-diving. This data could be really interesting, if confirmed by future in-depth specific analysis, and suggest that the mechanisms balancing the hyperbaric related oxidative stress are able to avoid the persistence of this condition at the end of the underwater phase of a dive.

In contrast, plasma levels of TBARS, did not show any change at the bottom and at the follow-up. A possible explanation is linked to the effectiveness of the activation of a complex endogenous antioxidant system (Ames et al., 1981; Stocker et al., 1987; Cao and Prior, 1998) to prevent the accumulation of free radicals and the subsequent damage of biological macromolecules. The main limitation of TBARS assay is thiobarbituric acid can react not only with MDA but also with aldehydes and ketones (final products of lipid peroxidation) that have similar MW and chemical structure, reducing the specify of the method. These compounds are often called MDA-like products. Despite the recurring criticism of the assay (Janero, 1990), this limitation haven’t deterred researcher groups from using the assay, as evidenced by over 1,300 entries over the last 10 years (using the search terms thiobarbituric acid and malondialdehyde or variants, e.g., TBA, MDA) in the Web of Science database (Ghani et al., 2017). Several methods have been proposed to increase the specificity of the TBA reaction to measure MDA (Giera et al., 2012; Papastergiadis et al., 2012; Diaz et al., 2014) but the spectrophotometric test remains the most common way to perform the assay: this has been also observed in some studies where TBARS assay seems to represent the one of best marker to assess the pro-oxidant/antioxidant equilibrium in a surgical context (Olszewska-Slonina et al., 2011; Jovanovic et al., 2019; Ottolenghi et al., 2019).

Actually, the current data on oxidative stress in underwater activities are controversial: the oxidative stress biomarkers do not behave the same way under hyperbaric/hyperoxic conditions. Some authors reported that BH-diving leads to an increase of TBARS levels while reduced glutathione (GSH) and reduced ascorbic acid (RAA) decrease (Joulia et al., 2003). Similar to SCUBA diving (Sureda et al., 2012), BH-divers can activate the endogenous antioxidant defenses to control vascular oxidative stress due to increased O2 level associated with hyperbaric conditions (Bulmer et al., 2008). Indeed, Bulmer et al. (2008) measured some antioxidant enzymes activities such as SOD, CAT, and GPx finding acute changes in these enzymes (especially SOD) suggesting that they may protect from excessive antioxidant depletion and oxidative stress during apnea. Finally, it is interesting to note that the data underwater related were not influenced by AGE and BMI.

## Study Limitations

This study represents a pilot test and has some limitations. First, the context where the tests were performed (the “Y-40 The Deep Joy” swimming pool) may have differences with respect to open water environments, especially regarding the temperature (33°C at our facility), but the Y-40 swimming pool currently represent the only feasible bench to perform such experiments.

TEAC assay is one of the most common methods to evaluate plasma TAC but its principal limitation is the non- specificity of the test. Despite this, TEAC assay is widely used to evaluate the plasma and serum TAC for its simplicity, solubility in aqueous media and suitability for automatic systems, permitting rapid throughput of samples and various kits based on this method are commercially available (Erel, 2004; Prior et al., 2005).

Similarly to TEAC method, the main limitation of TBARS assay is its non-specificity Other methods for measuring MDA, based on GC, HPLC (Del Rio et al., 2005), and capillary electrophoresis techniques (Wilson et al., 1997) have been developed to increase the specificity of the TBA reaction to measure just MDA, however, they have not yet gained widespread use, as the vast majority of workers still use the simple colorimetric test. For this reason, although not representing the cutting edge of sensitivity, the TBARS and TEAC assays still represent a reasonable compromise in terms of costs and reliability.

Finally, NO is involved in the activation of cGMP dependent signaling pathways in smooth muscle cells (Golshiri et al., 2020) and is strongly influenced by GSH redox equilibrium, further investigations of those pathways would be needed to look into our findings.

Furthermore, we may have been limited by the reduced sample size in this study.

## Conclusion and Prospectives

The increased plasma NOx values and the oxidative stress found at the bottom during BH-diving, were not observed immediately after reaching the surface, indicating a very rapid return to the pre-dive values. No lipid peroxidation was found. Our findings seem to indicate that NO availability after diving is not influenced by a NOx accumulation related to diving.

## Data Availability Statement

The raw data supporting the conclusions of this article will be made available by the authors, without undue reservation.

## Ethics Statement

The studies involving human participants were reviewed and approved by the Ethical Committee of the Università degli Studi di Milano, Italy (Aut. No. 37/17). The patients/participants provided their written informed consent to participate in this study.

## Author Contributions

DC implemented the systematic search strategy, extracted and analyzed the data, and wrote the first draft. AB was involved in the conception and design of this work, reviewed the critical appraisal of selected articles, and assisted with the compilation of the systematic review. MPi and MPa extracted and analyzed the data and reviews the manuscript. VL was involved in the test on the field and reviews the manuscript. MS, GB, and AM supervised the entire process. All the authors contributed to at least three of the four major components of a study and were involved in the conception and design of this work, contributed to the process of writing, and approval of the final manuscript.

## Conflict of Interest

The authors declare that the research was conducted in the absence of any commercial or financial relationships that could be construed as a potential conflict of interest.
